# 621. Self-inflicted Burn Injury: A Systematic Review and Meta-analysis

**DOI:** 10.1093/jbcr/irag033.435

**Published:** 2026-04-06

**Authors:** Diana B Griffin, Marlynn P Lopez, Emily Duckworth, Angelica Bartler, Aneeq Chaudhry, Tarifa Adam, Robert Galiano

**Affiliations:** Northwestern University, Chicago, IL; Northwestern University, Chicago, IL; University of South Carolina School of Medicine Greenville, Greenville, SC; Chicago Medical School - Rosalind Franklin University of Medicine and Science, Antioch, IL; Northwestern University, Chicago, IL; Northwestern University, Albany, NY; Northwestern University, Chicago, IL

## Abstract

**Introduction:**

Intentional burn injuries, while less common than unintentional burns, carry disproportionate psychosocial and clinical consequences and are often associated with high morbidity and mortality. We aim to understand and synthesize risk factors for self-immolation and burns intended to harm as well to evaluate the outcomes of these burns.

**Methods:**

A Prospero registration was created and literature search was conducted in September 2025, using the Embase, Ovid, PubMed, and Cochrane Library databases to identify observational studies involving adults who experienced burn injuries. Case reports, case series, reviews, editorials, letters to the editor, animal studies, and in vitro studies were excluded. Results were synthesized using random-effects models to generate pooled odds ratios (ORs) with 95% confidence intervals. Risk of bias assessments and sensitivity analysis were conducted. Analysis identified these comparators: self-inflicted burns (SIB) versus non-self-inflicted burns (NSIB), SIB versus burns by assault, intentional burns versus nonintentional burns, SIB versus unintentional burns, and SIB with suicidal intent versus non-suicidal SIB.

**Results:**

A total of 2654 articles were initially identified; 52 studies were selected for the systematic review and of these articles, 19 provided data sufficient for meta-analysis. Despite marked heterogeneity between studies, statistical analysis suggests that patients with SIB are more likely to have a prior psychiatric history compared to patients with NSIB (OR 28.54, 95% CI 11.18-72.86). Patients with SIB also have higher odds of mortality than those with NSIB (OR 5.15, 95% CI 3.18-8.36). SIB have increased odds of inhalation injury compared to NSIB (OR 4.53, 95% CI 2.08-9.87), burns by assault (OR 3.17, 95% CI 1.00-10.03), and unintentional burns (OR 4.09, 95% CI 2.59-6.50). Intubation with ICU admission is at greater odds with SIB relative to NSIB (OR 3.46, 95% CI 0.67-17). No significant differences in type of burn (i.e., chemical, open flame, etc.) or body site were found among any comparators.

**Conclusions:**

This study highlights key psychiatric factors associated with SIB and intentional burns and points to possible associations with worse outcomes and higher mortality. Data collection and assessment that further investigates burn characteristics such as burn degree, TBSA, length of stay in hospital, or burn site could demonstrate that SIB are more severe than its counterparts, supporting the association with increased mortality; however, for this review data found was too limited.

**Applicability of Research to Practice:**

We hope a more holistic understanding of SIB and burns with intent to harm self or others can inform the development of targeted burn prevention efforts and inform interdisciplinary clinical management to simultaneously optimize wound management and mental health.

**Funding for the study:**

N/A.

